# The Role of the P2X7 Receptor in Ocular Stresses: A Potential Therapeutic Target

**DOI:** 10.3390/vision1020014

**Published:** 2017-05-17

**Authors:** Mélody Dutot, Elodie Olivier, Anaïs Wakx, Patrice Rat

**Affiliations:** 1 UMR 8638 CNRS COMETE, Université Paris Descartes, Sorbonne Paris Cité, Faculté de Pharmacie, 4 Avenue de l’Observatoire, 75006 Paris, France; 2 Recherche et Développement, Laboratoire d’Evaluation Physiologique, Yslab, 2 rue Félix Le Dantec, 29000 Quimper, France

**Keywords:** ocular, stress, P2X7 receptor, modulation, therapeutic target

## Abstract

The P2X7 receptor is expressed in both anterior and posterior segments of the eyeball. In the ocular surface, the P2X7 receptor is activated in case of external aggressions: preservatives and surfactants induce the activation of P2X7 receptors, leading to either apoptosis, inflammation, or cell proliferation. In the retina, the key endogenous actors of age-related macular degeneration, diabetic retinopathy, and glaucoma act through P2X7 receptors’ activation and/or upregulation of P2X7 receptors’ expression. Different therapeutic strategies aimed at the P2X7 receptor exist. P2X7 receptor antagonists, such as divalent cations and Brilliant Blue G (BBG) could be used to target either the ocular surface or the retina, as long as polyunsaturated fatty acids may exert their effects through the disruption of plasma membrane lipid rafts or saffron that reduces the response evoked by P2X7 receptor stimulation. Treatments against P2X7 receptor activation are proposed by using either eye drops or food supplements.

## 1. Introduction

The purinergic P2X family is composed of ionotropic receptors; seven receptor subtypes have been identified (P2X1 to P2X7). The P2X7 receptor, formerly also known as P2Z, is ubiquitously expressed in a wide variety of cell types including cells of haematopoietic origin (mast cells, macrophages, fibroblasts, erythrocytes, granulocytes, erythroleukaemia cells, and lymphocytes), central and spinal cord neurons, brain glial cells (microglia, astrocytes, and Müller cells), bone cells (osteoblasts, osteoclasts, and osteocytes), and epithelial and endothelial cells [1,2,3,4,5,6,7,8,9,10]. The P2X7 receptor has also been detected in both anterior and posterior segments of the eyeball: cornea and conjunctiva were immunopositive [11,12,13,14], as were the different layers of the retina [11,15,16,17,18,19], lacrimal glands [20], and lens cells [11,21] (see Table 1 for more details). The P2X7 receptor is highly polymorphic and nine different human splice variants have been identified [22].

The P2X7 receptor is restrictively activated by ATP^4−^, requiring higher concentrations of ATP to be activated when compared with other P2X purinoreceptors. The P2X7 receptor is sensitive to a few nucleotides apart from BzATP (2′(3′)-O-(4-Benzoylbenzoyl)adenosine 5′-triphosphate triethylammonium salt), which is 10 to 100 times more potent than ATP [23,24].

P2X7 receptors show complex gating behavior: several seconds of ATP exposure induces P2X7 receptors’ dilatation from a channel that allows for the passage of small cations to a pore that allows for permeation of larger cations and dyes such as YO-PRO-1 (see YO-PRO-1 staining protocol in [25]). P2X7 receptor activation triggers numerous cellular effects from oxidative stress to apoptosis, including inflammation. As previously described, P2X7 receptors activate apoptotic caspases 3, 8, and 9 [26,27], and are involved in actin reorganization and plasma membrane blebs formation through p38, Mitogen-Activated Protein Kinases (MAPK), and Rho-GTPases [28]. The activation of P2X7 receptors can also induce an inflammatory response through the formation of the inflammasome complex, which leads to proinflammatory cytokine release [26,29,30,31,32,33,34,35,36]. All these toxic cellular events occur during the pathogenesis of degenerative disorders, which highlights the pivotal role of P2X7 receptors in these diseases. It is worth mentioning that P2X7 receptors are also involved in life signals such as cell proliferation [37,38]. This effect on proliferation has been associated to wound healing when P2X7 receptor activation is induced [39] but also to cancer when P2X7 receptor expression is increased, allowing cancerous cell survival and proliferation [40,41]. The proliferative effects triggered by P2X7 receptor activation may be elicited at basal or low ATP concentrations [42], and/or depends on isoform expression [43].

P2X7 receptors are of great interest to toxicologists because as membrane receptors, they can be easily targeted by therapeutic formulations to block the toxic mechanisms they trigger. This review will be dedicated to the implication of P2X7 receptors in ocular toxic stresses and the existing modulators that are or could be used in ophthalmology.

## 2. P2X7 Receptor Activation in the Case of Ocular Stresses

### 2.1. Exogenous Stresses: Chemical and Mechanical Injuries

The ocular surface is vulnerable to potential environmental stresses by the nature of its function and anatomic location. This section will focus on the role of P2X7 receptors in the main exogenous stresses impacting the ocular surface.

#### 2.1.1. Preservatives

Despite their well-known toxicity for the ocular surface, preservatives are still present in numerous topical ocular medications. The quaternary ammonium benzalkonium chloride (BAC) would be the most frequently used preservative for preparations, such as multidose eye drops, that require the inclusion of an antimicrobial preservative. Preserved multidose eye drops are generally used for long-term treatments such as glaucoma or in case of ocular infections that need long-term antibiotic treatment and repeated treatments (sometimes more than eight times a day). It has now been clearly demonstrated that BAC can induce ocular discomfort, dry eye, itching, or foreign body sensation [44,45]. Those symptoms have been linked, inter alia, to the apoptosis of corneal and conjunctival cells [46,47,48]. We have demonstrated that BAC, at the same concentrations as those used in eye drops (0.0025–0.01%), activates the P2X7 receptor as a consequence of ATP release, leading to the death of corneal and conjunctival cells [11,49,50]. Preserved latanoprost, travoprost, and bimatoprost solutions, all of them being antiglaucoma prostaglandin analog eye drops, induced the activation of P2X7 receptors (+130% to +400%) in conjunctival cells [50] that could partially contribute to inflammatory stimulation throughout the ocular surface in glaucoma patients under treatment. Preserved ofloxacin fluoroquinolone eye drops induced high activation of P2X7 receptors (+900% to +5500%) on ocular surface cells [49]. In that case, the P2X7 receptor acts as a P2Z/P2X7 cytolytic receptor, which may explain the corneal perforations observed after repeated treatment with preserved fluoroquinolone eye drops [51].

In the field of contactology, preservatives are widely used to clean contact lenses using multipurpose solutions. Polidronium chloride (a quaternary ammonium) and polyhexamethylene biguanides (PHMB) are the most commonly used preservatives in contactology. Despite the very low concentrations of preservatives present in multipurpose solutions (polidronium chloride around 0.001%, and PHMB between 0.00005% and 0.0001%), P2X7 receptors were activated on ocular surface cells after a short incubation time [52,53]. Apoptosis induced by multipurpose solutions can lead to contact lens intolerance over time; that is why eye care professionals recommend the use of a supplemental rinse step of contact lenses with unpreserved saline solutions [54,55,56].

#### 2.1.2. Surfactants

Surfactants may be included in ophthalmic suspensions to disperse the drug effectively during manufacturing and product use. They are also used to stabilize emulsions that are generally prepared by dissolving or dispersing lipophilic active ingredients into an oil phase by adding suitable emulsifying agents and mixing with water to form oil-in-water emulsions. Nevertheless, excessive amounts can lead to irritation in the eye [57,58]. Sodium lauryl sulfate (SLS) is an anionic surfactant used in cosmetics (toothpastes, shampoos, shaving foams, etc.), and yet it is classified as an irritant product. Pauloin et al., indicated that SLS activates P2X7 receptors using an in vitro corneal cell model and that these P2X7 toxic effects can be inhibited by high-molecular weight hyaluronan [59]. Polysorbates are used in lubricant eye drops to help reduce tear evaporation by stabilizing the lipid layer. They are also widely used, as well as caprylocaproyl polyoxylglycerides (Labrasol™) and polyethoxylated castor oil (Cremophor™ EL), in nanoemulsions for ophthalmic preparations to stabilize lipophilic active ingredients such as ciclosporin A [60]. Nevertheless, the cytotoxic effects they exert on ocular surface cells [61,62] may limit their use. We showed that castor oil, caprylocaproyl polyoxylglyceride, and polysorbate 85 all induced P2X7 receptor activation in conjunctival cells [63,64].

#### 2.1.3. Trauma

Corneal abrasion is one of the most common eye injuries. The causes are as numerous as they are different: tree branches, makeup brushes, a finger, a pet, workplace debris, sports equipment, sand, dust, etc. The main consequences of corneal abrasions are significant discomfort, red eyes, and photophobia [65], and complications can be severe and may lead to blindness if not treated correctly. Corneal abrasions lead to the loss of corneal cells, which makes the eye more susceptible to infection. Therefore, wound healing in the cornea is essential for maintaining the health of the ocular tissue and preventing pathologies. The early response after injury is critical as it initiates essential signaling pathways required for proper wound healing. The P2X7 receptor is necessary for the healing of abrasion wounds by promoting epithelial cell adhesion to the basement membrane coordinating Ca^2+^ mobilization, cytoskeletal rearrangement, and normal stromal collagen structure [12,13]. Interestingly, in P2X7^−/−^ mice, the downward trend in the rate of epithelial wound repair was associated to deleterious morphologic changes at the leading edge of the wound, compared to control mice. Both P2X7 and P2Y2 receptors are necessary for proper corneal wound repair, with P2X7 receptors coordinating signals along the wound margin and P2Y2 coordinating signals back from the wound. Mankus et al. observed that the P2X7 receptor in the corneal epithelium is expressed in both full-length and variant forms and displays different functions [66]. The variant P2X7 receptor may be responsible for corneal tissue flexibility: it is mainly expressed in corneal cells that migrate and become stratified during wound healing. More recently, it has been shown that P2Y2 receptor stimulation supplemented with a P2X7 agonist such as BzATP can improve corneal wound healing outcomes [67].

### 2.2. Endogenous Stresses: Biological Stresses

An endogenous stress takes its origin from the inside of the organism. The P2X7 receptor is related to numerous biological endogenous stresses that occur in the eye, especially in the retina. Among these stresses, we can highlight the main actors of age-related macular degeneration (AMD) such as amyloid β and oxysterols, high glucose inducing diabetic retinopathy, and increased hydrostatic pressure leading to glaucoma.

#### 2.2.1. AMD

AMD is one of the most common causes of severe vision loss worldwide. Proteolipidic deposits called drusen, formed in the retina, characterize AMD. Amyloid β, a highly toxic peptide, is found in drusen [68,69]. We demonstrated that the P2X7 receptor plays a key role in amyloid β-induced degeneration of retinal cells [70]. Amyloid β lead to apoptosis, a hallmark of retinal degeneration [71], via P2X7 receptor activation in human retinal Müller glial cells. The P2X7 receptor is also involved in microglia activation and alterations induced by amyloid β [72,73,74].

Oxysterols, oxidized derivatives of cholesterol, are known for their involvement in degenerative diseases [75,76]. In AMD, oxysterols accumulate in drusen and have been associated with the retinal degeneration process [77,78,79]. We recently highlighted the key role of P2X7 receptor activation in oxysterol-induced retinal degeneration in human retinal pigmented epithelial cells [80].

#### 2.2.2. Diabetic Retinopathy

A high level of serum glucose triggers diabetes and ophthalmic complications such as diabetic retinopathy, which is characterized by alterations of retinal blood vessels. A high glucose concentration induced ATP-mediated apoptosis through P2X7 receptor activation in human fibroblasts [81]. In retinal neurons and microglia, intracellular calcium increased after purinergic stimulation [82]. In the case of diabetes, retinal microvessels are more sensitive to P2X7 receptor activation, meaning that for the same activation of P2X7 receptors, cellular effects and particularly apoptosis were increased in retinal microvessels exposed to high glucose concentrations [83].

#### 2.2.3. Glaucoma

Glaucoma is a major cause of blindness worldwide. An increase in intraocular pressure alters the optic nerve, leading to vision loss. Acute elevation of intraocular pressure leads to an increase in P2X7 receptor expression in retinal ganglion cells [84]. Besides, neuronal mechanical deformations that occur after changes in intraocular pressure induced ATP release in retinal ganglion cells, triggering P2X7 receptor stimulation [85]. Stimulation of the P2X7 receptor then mediated retinal ganglion cell death, which plays a role in ischemia-induced neurodegeneration in the human retina [86]. More generally, ATP-induced activation of P2X7 receptors contributes to the pathogenesis of glaucoma [87].

## 3. Anti-P2X7 Strategies in Ophthalmology

The P2X7 receptor has received particular attention as a potential therapeutic target because of its widespread involvement in numerous ocular diseases. It appears to be a key regulatory element of apoptosis, inflammation, and cell death in general. Several P2X7 receptor antagonists have been evaluated in ophthalmology, and both topical and oral administrations have been considered.

### 3.1. Topical Administration

Topical administration, mostly in the form of eye drops, is employed to treat diseases of the anterior segment, usually the cornea and the conjunctiva. Hyaluronan is a natural polysaccharide used in ophthalmology in artificial tears for the treatment of dry eye syndrome [88,89,90,91], due to its lubricant and viscoelastic properties. Hyaluronan has also been considered as a potent P2X7 receptor modulator: a pretreatment with hyaluronan before BAC or SLS incubation was able to significantly decrease SLS-induced P2X7 activation in corneal and conjunctival cells [59,92,93]. One possible mechanism is that hyaluronan physically coats the cell membrane via strong links with CD44 receptors. At the same time, it masks P2X7 receptors, preventing their activation.

The P2X7 receptor is potently inhibited by divalent cations such as calcium, magnesium, zinc, and copper, that on the one hand alter the affinity of ATP binding to the P2X7 receptor in an allosteric manner, and on the other hand directly interact with the P2X7 receptor [94,95,96,97,98]. It has been demonstrated that some marine solutions containing Ca^2+^, Mg^2+^, and Zn^2+^ inhibited basal activation of P2X7 receptors in ocular surface cell lines [11]. Such solutions that are rich in divalent cations could be easily used as P2X7 receptor modulators or to potentiate P2X7 receptor antagonists.

### 3.2. Oral Administration

The oral route of drug administration to target the eye may not be the most efficient delivery system due to absorption from the gastrointestinal tract and high systemic clearance rates. Nevertheless, oral antioxidants are prescribed to dry AMD patients since no truly effective treatment is currently available for patients with advanced disease. A proof-of-principle clinical trial in AMD patients confirmed the positive effects of antioxidant saffron administration in neurodegenerative diseases and its persistence over time [99,100]. Recent data showed that saffron may exert its protective role in neurodegeneration by reducing the intracellular calcium response evoked by P2X7 receptor stimulation [101].

Omega-3 polyunsaturated fatty acids were included in the formulation used in the Second Age-Related Eye Disease Study (AREDS2) to evaluate their effects on slowing the progression of AMD [102]. The results of AREDS2 showed that omega-3 fatty acid supplementation did not yield a statistically significant reduction in the progression of AMD [103]. However, previous observational studies suggested a link between high dietary consumption of omega-3 fatty acids and decreased risk of developing advanced AMD [104,105]. We demonstrated that eicosapentaenoic acid (EPA) and docosahexaenoic acid (DHA) represent efficient modulators of amyloid β toxicity in retinal cells with the condition that these omega-3 fatty acids are brought as triglycerides in fish oils, and not as ethyl esters like in AREDS2 [70]. The preventive effects of fish EPA and DHA against amyloid β-induced apoptosis relies on the inhibition of P2X7 receptors through lipid raft disruption. In combination with Brilliant Blue G (BBG), a specific P2X7 receptor inhibitor, they fully prevented amyloid β cytotoxic effects. It was then concluded that marine oils rich in EPA and DHA plus BBG in food supplements could be proposed to prevent AMD. Other studies determined the promising role of BBG as a therapeutic agent to inhibit AMD expansion, as it might prevent retinal pigmented epithelium and photoreceptor cell death [106,107,108].

## 4. Conclusions

Excessive ATP release is implicated in numerous ocular stresses. As the P2X7 receptor is expressed in the whole eye, it plays a key role in ATP-induced mechanisms, whether they are triggered in the anterior segment or the posterior segment, and whether they are induced by exogenous chemicals such as preservatives or by endogenous agents such as amyloid β. Consequently, P2X7 receptor inhibition is considered as a potent therapeutic strategy and several P2X7 receptor antagonists could be used in the ophthalmology field, but further clinical studies are necessary to clearly identify efficient and bioavailable ophthalmic formulations.

## Figures and Tables

**Table 1:** Expression of the P2X7 receptor in the eye.

Tissue	Cells	mRNA	Protein	Species	References
Cornea	Corneal epithelial cells (Human Corneal Epithelial (HCE) cell line)		+	Human	[11]
	Cornea section		+	Mouse	[12]
	Corneolimbal epithelial cells (telomerase immortalized cells)	+	+	Human	[66,109]
Conjunctiva	Conjunctival epithelial cells		+	Human	[109]
	Conjunctival epithelial cells (Wong-Kilbourne derivative of Chang conjunctiva (WKD) cell line)		+	Human	[11]
	Goblet cells		+	Rat	[14]
Lens	Lens fiber cells	+	+	Rat	[21]
	Lens epithelial cells		+	Human	[11]
Retina	Müller glial cells	+	+	Human	[18,70]
	Retina section	+	+	Rat	[110]
	Retinal ganglion cells	+		Rat	[111]
	Retinal pigmented epithelial cells (Acute Retinal Pigment Epithelial-19 (ARPE-19) cell line)		+	Human	[11]
	Retinal pigmented epithelial cells (primary culture)	+	+	Human	[112]
	Retinal pigmented epithelial cells	+	+	Mouse	[113]
	Photoreceptors	+	+	Rat	[114]
Ciliary body	Ciliary epithelial cells	+		Bovine	[115]

## References

[1] North R.A. (2002). Molecular physiology of P2X receptors. Physiol. Rev..

[2] Ralevic V., Burnstock G. (1998). Receptors for purines and pyrimidines. Pharmacol. Rev..

[3] Lundy P.M., Hamilton M.G., Mi L., Gong W., Vair C., Sawyer T.W., Frew R. (2002). Stimulation of Ca(^2+^) influx through ATP receptors on rat brain synaptosomes: Identification of functional P2X(7) receptor subtypes. Br. J. Pharmacol..

[4] Miras-Portugal M.T., Diaz-Hernandez M., Giraldez L., Hervas C., Gomez-Villafuertes R., Sen R.P., Gualix J., Pintor J. (2003). P2X7 receptors in rat brain: Presence in synaptic terminals and granule cells. Neurochem. Res..

[5] Deuchars S.A., Atkinson L., Brooke R.E., Musa H., Milligan C.J., Batten T.F., Buckley N.J., Parson S.H., Deuchars J. (2001). Neuronal P2X7 receptors are targeted to presynaptic terminals in the central and peripheral nervous systems. J. Neurosci..

[6] Li J., Liu D., Ke H.Z., Duncan R.L., Turner C.H. (2005). The P2X7 nucleotide receptor mediates skeletal mechanotransduction. J. Biol. Chem..

[7] Jorgensen N.R., Henriksen Z., Sorensen O.H., Eriksen E.F., Civitelli R., Steinberg T.H. (2002). Intercellular calcium signaling occurs between human osteoblasts and osteoclasts and requires activation of osteoclast P2X7 receptors. J. Biol. Chem..

[8] Li Q., Luo X., Zeng W., Muallem S. (2003). Cell-specific behavior of P2X7 receptors in mouse parotid acinar and duct cells. J. Biol. Chem..

[9] Bardini M., Lee H.Y., Burnstock G. (2000). Distribution of P2X receptor subtypes in the rat female reproductive tract at late pro-oestrus/early oestrus. Cell Tissue Res..

[10] Wang Q., Wang L., Feng Y.H., Li X., Zeng R., Gorodeski G.I. (2004). P2X7 receptor-mediated apoptosis of human cervical epithelial cells. Am. J. Physiol. Cell Physiol..

[11] Dutot M., Liang H., Pauloin T., Brignole-Baudouin F., Baudouin C., Warnet J.M., Rat P. (2008). Effects of toxic cellular stresses and divalent cations on the human P2X7 cell death receptor. Mol. Vis..

[12] Mayo C., Ren R., Rich C., Stepp M.A., Trinkaus-Randall V. (2008). Regulation by P2X7: Epithelial migration and stromal organization in the cornea. Investig. Ophthalmol. Vis. Sci..

[13] Minns M.S., Teicher G., Rich C.B., Trinkaus-Randall V. (2016). Purinoreceptor P2X7 Regulation of Ca(^2+^) Mobilization and Cytoskeletal Rearrangement Is Required for Corneal Reepithelialization after Injury. Am. J. Pathol..

[14] McGilligan V.E., Gregory-Ksander M.S., Li D., Moore J.E., Hodges R.R., Gilmore M.S., Moore T.C., Dartt D.A. (2013). Staphylococcus aureus activates the NLRP3 inflammasome in human and rat conjunctival goblet cells. PLoS ONE.

[15] Bowes Rickman C., Farsiu S., Toth C.A., Klingeborn M. (2013). Dry age-related macular degeneration: Mechanisms, therapeutic targets, and imaging. Investig. Ophthalmol. Vis. Sci..

[16] Ambati J., Atkinson J.P., Gelfand B.D. (2013). Immunology of age-related macular degeneration. Nat. Rev. Immunol..

[17] Kauppinen A., Paterno J.J., Blasiak J., Salminen A., Kaarniranta K. (2016). Inflammation and its role in age-related macular degeneration. Cell. Mol. Life Sci..

[18] Pannicke T., Fischer W., Biedermann B., Schadlich H., Grosche J., Faude F., Wiedemann P., Allgaier C., Illes P., Burnstock G. (2000). P2X7 receptors in Muller glial cells from the human retina. J. Neurosci..

[19] Vessey K.A., Fletcher E.L. (2012). Rod and cone pathway signalling is altered in the P2X7 receptor knock out mouse. PLoS ONE.

[20] Hodges R.R., Vrouvlianis J., Shatos M.A., Dartt D.A. (2009). Characterization of P2X7 purinergic receptors and their function in rat lacrimal gland. Investig. Ophthalmol. Vis. Sci..

[21] Suzuki-Kerr H., Vlajkovic S., Donaldson P.J., Lim J. (2008). Molecular identification and localization of P2X receptors in the rat lens. Exp. Eye Res..

[22] Sluyter R., Stokes L. (2011). Significance of P2X7 receptor variants to human health and disease. Recent Pat. DNA Gene Seq..

[23] Dubyak G.R., el-Moatassim C. (1993). Signal transduction via P2-purinergic receptors for extracellular ATP and other nucleotides. Am. J. Physiol..

[24] Di Virgilio F. (1995). The P2Z purinoceptor: An intriguing role in immunity, inflammation and cell death. Immunol. Today.

[25] Rat P., Olivier E., Tanter C., Wakx A., Dutot M. (2017). A fast and reproducible cell- and 96-well plate-based method for the evaluation of P2X7 receptor activation using YO-PRO-1 fluorescent dye. J. Biol. Methods.

[26] Ferrari D., Los M., Bauer M.K., Vandenabeele P., Wesselborg S., Schulze-Osthoff K. (1999). P2Z purinoreceptor ligation induces activation of caspases with distinct roles in apoptotic and necrotic alterations of cell death. FEBS Lett..

[27] Kong Q., Wang M., Liao Z., Camden J.M., Yu S., Simonyi A., Sun G.Y., Gonzalez F.A., Erb L., Seye C.I. (2005). P2X(7) nucleotide receptors mediate caspase-8/9/3-dependent apoptosis in rat primary cortical neurons. Purinergic Signal..

[28] Pfeiffer Z.A., Aga M., Prabhu U., Watters J.J., Hall D.J., Bertics P.J. (2004). The nucleotide receptor P2X7 mediates actin reorganization and membrane blebbing in RAW 264.7 macrophages via p38 MAP kinase and Rho. J. Leukoc. Biol..

[29] Ferrari D., Wesselborg S., Bauer M.K., Schulze-Osthoff K. (1997). Extracellular ATP activates transcription factor NF-kappaB through the P2Z purinoreceptor by selectively targeting NF-kappaB p65. J. Cell Biol..

[30] Ferrari D., Pizzirani C., Adinolfi E., Lemoli R.M., Curti A., Idzko M., Panther E., Di Virgilio F. (2006). The P2X7 receptor: A key player in IL-1 processing and release. J. Immunol..

[31] Labasi J.M., Petrushova N., Donovan C., McCurdy S., Lira P., Payette M.M., Brissette W., Wicks J.R., Audoly L., Gabel C.A. (2002). Absence of the P2X7 receptor alters leukocyte function and attenuates an inflammatory response. J. Immunol..

[32] Pelegrin P., Barroso-Gutierrez C., Surprenant A. (2008). P2X7 receptor differentially couples to distinct release pathways for IL-1beta in mouse macrophage. J. Immunol..

[33] Solini A., Chiozzi P., Morelli A., Fellin R., Di Virgilio F. (1999). Human primary fibroblasts in vitro express a purinergic P2X7 receptor coupled to ion fluxes, microvesicle formation and IL-6 release. J. Cell Sci..

[34] Solle M., Labasi J., Perregaux D.G., Stam E., Petrushova N., Koller B.H., Griffiths R.J., Gabel C.A. (2001). Altered cytokine production in mice lacking P2X(7) receptors. J. Biol. Chem..

[35] Franceschini A., Capece M., Chiozzi P., Falzoni S., Sanz J.M., Sarti A.C., Bonora M., Pinton P., Di Virgilio F. (2015). The P2X7 receptor directly interacts with the NLRP3 inflammasome scaffold protein. FASEB J..

[36] Kahlenberg J.M., Dubyak G.R. (2004). Mechanisms of caspase-1 activation by P2X7 receptor-mediated K^+^ release. Am. J. Physiol. Cell Physiol..

[37] Baricordi O.R., Ferrari D., Melchiorri L., Chiozzi P., Hanau S., Chiari E., Rubini M., Di Virgilio F. (1996). An ATP-activated channel is involved in mitogenic stimulation of human T lymphocytes. Blood.

[38] Baricordi O.R., Melchiorri L., Adinolfi E., Falzoni S., Chiozzi P., Buell G., Di Virgilio F. (1999). Increased proliferation rate of lymphoid cells transfected with the P2X(7) ATP receptor. J. Biol. Chem..

[39] Ghazi K., Deng-Pichon U., Warnet J.M., Rat P. (2012). Hyaluronan fragments improve wound healing on in vitro cutaneous model through P2X7 purinoreceptor basal activation: Role of molecular weight. PLoS ONE.

[40] Adinolfi E., Raffaghello L., Giuliani A.L., Cavazzini L., Capece M., Chiozzi P., Bianchi G., Kroemer G., Pistoia V., Di Virgilio F. (2012). Expression of P2X7 receptor increases in vivo tumor growth. Cancer Res..

[41] Giannuzzo A., Pedersen S.F., Novak I. (2015). The P2X7 receptor regulates cell survival, migration and invasion of pancreatic ductal adenocarcinoma cells. Mol. Cancer.

[42] Adinolfi E., Pizzirani C., Idzko M., Panther E., Norgauer J., Di Virgilio F., Ferrari D. (2005). P2X(7) receptor: Death or life?. Purinergic Signal..

[43] Adinolfi E., Cirillo M., Woltersdorf R., Falzoni S., Chiozzi P., Pellegatti P., Callegari M.G., Sandona D., Markwardt F., Schmalzing G. (2010). Trophic activity of a naturally occurring truncated isoform of the P2X7 receptor. FASEB J..

[44] Pisella P.J., Pouliquen P., Baudouin C. (2002). Prevalence of ocular symptoms and signs with preserved and preservative free glaucoma medication. Br. J. Ophthalmol..

[45] Jaenen N., Baudouin C., Pouliquen P., Manni G., Figueiredo A., Zeyen T. (2007). Ocular symptoms and signs with preserved and preservative-free glaucoma medications. Eur. J. Ophthalmol..

[46] Baudouin C., Labbe A., Liang H., Pauly A., Brignole-Baudouin F. (2010). Preservatives in eyedrops: The good, the bad and the ugly. Prog. Retinal Eye Res..

[47] Chen W., Dong N., Huang C., Zhang Z., Hu J., Xie H., Pan J., Liu Z. (2014). Corneal alterations induced by topical application of commercial latanoprost, travoprost and bimatoprost in rabbit. PLoS ONE.

[48] Kim E.J., Kim Y.H., Kang S.H., Lee K.W., Park Y.J. (2013). In vitro effects of preservative-free and preserved prostaglandin analogs on primary cultured human conjunctival fibroblast cells. Korean J. Ophthalmol..

[49] Dutot M., Pouzaud F., Larosche I., Brignole-Baudouin F., Warnet J.M., Rat P. (2006). Fluoroquinolone eye drop-induced cytotoxicity: Role of preservative in P2X7 cell death receptor activation and apoptosis. Investig. Ophthalmol. Vis. Sci..

[50] Brasnu E., Brignole-Baudouin F., Riancho L., Guenoun J.M., Warnet J.M., Baudouin C. (2008). In vitro effects of preservative-free tafluprost and preserved latanoprost, travoprost, and bimatoprost in a conjunctival epithelial cell line. Curr. Eye Res..

[51] Mallari P.L.T., McCarty D.J., Daniell M., Taylor H. (2001). Increased incidence of corneal perforation after topical fluoroquinolone treatment for microbial keratitis. Am. J. Ophthalmol..

[52] Dutot M., Warnet J.M., Baudouin C., Rat P. (2008). Cytotoxicity of contact lens multipurpose solutions: Role of oxidative stress, mitochondrial activity and P2X7 cell death receptor activation. Eur. J. Pharm. Sci..

[53] Dutot M., Paillet H., Chaumeil C., Warnet J.M., Rat P. (2009). Severe ocular infections with contact lens: Role of multipurpose solutions. Eye (Lond.).

[54] Dutot M., Vincent J., Martin-Brisac N., Fabre I., Grasmick C., Rat P. (2013). Ocular cytotoxicity evaluation of medical devices such as contact lens solutions and benefits of a rinse step in cleaning procedure. Altex.

[55] Choy C.K., Cho P., Boost M.V. (2013). Cytotoxicity of rigid gas-permeable lens care solutions. Clin. Exp. Optom..

[56] Choy C.K., Cho P., Boost M.V. (2012). Cytotoxicity and effects on metabolism of contact lens care solutions on human corneal epithelium cells. Clin. Exp. Optom..

[57] Cater K.C., Harbell J.W. (2006). Prediction of eye irritation potential of surfactant-based rinse-off personal care formulations by the bovine corneal opacity and permeability (BCOP) assay. Cutan. Ocul. Toxicol..

[58] Tachon P., Cotovio J., Dossou K.G., Prunieras M. (1989). Assessment of surfactant cytotoxicity: Comparison with the Draize eye test. Int. J. Cosmet. Sci..

[59] Pauloin T., Dutot M., Liang H., Chavinier E., Warnet J.M., Rat P. (2009). Corneal protection with high-molecular-weight hyaluronan against in vitro and in vivo sodium lauryl sulfate-induced toxic effects. Cornea.

[60] Lee S.I., Yang J.S., Lee G.H., Choi B.S., Ryu J.H. (2013). Nanoemulsion-type ophthalmic composition.

[61] Burgalassi S., Chetoni P., Monti D., Saettone M.F. (2001). Cytotoxicity of potential ocular permeation enhancers evaluated on rabbit and human corneal epithelial cell lines. Toxicol. Lett..

[62] Ujhelyi Z., Fenyvesi F., Varadi J., Feher P., Kiss T., Veszelka S., Deli M., Vecsernyes M., Bacskay I. (2012). Evaluation of cytotoxicity of surfactants used in self-micro emulsifying drug delivery systems and their effects on paracellular transport in Caco-2 cell monolayer. Eur. J. Pharm. Sci..

[63] Said T., Dutot M., Christon R., Beaudeux J.L., Martin C., Warnet J.M., Rat P. (2007). Benefits and side effects of different vegetable oil vectors on apoptosis, oxidative stress, and P2X7 cell death receptor activation. Investig. Ophthalmol. Vis. Sci..

[64] Sigward E., Mignet N., Rat P., Dutot M., Muhamed S., Guigner J.M., Scherman D., Brossard D., Crauste-Manciet S. (2013). Formulation and cytotoxicity evaluation of new self-emulsifying multiple W/O/W nanoemulsions. Int. J. Nanomed..

[65] Jayamanne D.G., Fitt A.W., Dayan M., Andrews R.M., Mitchell K.W., Griffiths P.G. (1997). The effectiveness of topical diclofenac in relieving discomfort following traumatic corneal abrasions. Eye.

[66] Mankus C., Rich C., Minns M., Trinkaus-Randall V. (2011). Corneal epithelium expresses a variant of P2X(7) receptor in health and disease. PLoS ONE.

[67] Minns M.S., Trinkaus-Randall V. (2016). Purinergic Signaling in Corneal Wound Healing: A Tale of 2 Receptors. J. Ocul. Pharmacol. Ther..

[68] Bruban J., Glotin A.L., Dinet V., Chalour N., Sennlaub F., Jonet L., An N., Faussat A.M., Mascarelli F. (2009). Amyloid-beta(1–42) alters structure and function of retinal pigmented epithelial cells. Aging Cell.

[69] Ratnayaka J.A., Serpell L.C., Lotery A.J. (2015). Dementia of the eye: The role of amyloid beta in retinal degeneration. Eye (Lond.).

[70] Wakx A., Dutot M., Massicot F., Mascarelli F., Limb G.A., Rat P. (2016). Amyloid beta Peptide Induces Apoptosis Through P2X7 Cell Death Receptor in Retinal Cells: Modulation by Marine Omega-3 Fatty Acid DHA and EPA. Appl. Biochem. Biotechnol..

[71] Dunaief J.L., Dentchev T., Ying G.S., Milam A.H. (2002). The role of apoptosis in age-related macular degeneration. Arch. Ophthalmol..

[72] Parvathenani L.K., Tertyshnikova S., Greco C.R., Roberts S.B., Robertson B., Posmantur R. (2003). P2X7 mediates superoxide production in primary microglia and is up-regulated in a transgenic mouse model of Alzheimer’s disease. J. Biol. Chem..

[73] McLarnon J.G., Ryu J.K., Walker D.G., Choi H.B. (2006). Upregulated expression of purinergic P2X(7) receptor in Alzheimer disease and amyloid-beta peptide-treated microglia and in peptide-injected rat hippocampus. J. Neuropathol. Exp. Neurol..

[74] Ni J., Wang P., Zhang J., Chen W., Gu L. (2013). Silencing of the P2X(7) receptor enhances amyloid-beta phagocytosis by microglia. Biochem. Biophys. Res. Commun..

[75] Lordan S., Mackrill J.J., O’Brien N.M. (2009). Oxysterols and mechanisms of apoptotic signaling: Implications in the pathology of degenerative diseases. J. Nutr. Biochem..

[76] Zarrouk A., Vejux A., Mackrill J., O’Callaghan Y., Hammami M., O’Brien N., Lizard G. (2014). Involvement of oxysterols in age-related diseases and ageing processes. Ageing Res. Rev..

[77] Javitt N.B., Javitt J.C. (2009). The retinal oxysterol pathway: A unifying hypothesis for the cause of age-related macular degeneration. Curr. Opin. Ophthalmol..

[78] Rodriguez I.R., Clark M.E., Lee J.W., Curcio C.A. (2014). 7-ketocholesterol accumulates in ocular tissues as a consequence of aging and is present in high levels in drusen. Exp. Eye Res..

[79] Rodriguez I.R., Larrayoz I.M. (2010). Cholesterol oxidation in the retina: Implications of 7KCh formation in chronic inflammation and age-related macular degeneration. J. Lipid Res..

[80] Olivier E., Dutot M., Regazzetti A., Leguillier T., Dargere D., Auzeil N., Laprevote O., Rat P. (2016). P2X7-pannexin-1 and amyloid beta-induced oxysterol input in human retinal cell: Role in age-related macular degeneration?. Biochimie.

[81] Solini A., Chiozzi P., Falzoni S., Morelli A., Fellin R., Di Virgilio F. (2000). High glucose modulates P2X7 receptor-mediated function in human primary fibroblasts. Diabetologia.

[82] Pereira Tde O., da Costa G.N., Santiago A.R., Ambrosio A.F., dos Santos P.F. (2010). High glucose enhances intracellular Ca^2+^ responses triggered by purinergic stimulation in retinal neurons and microglia. Brain Res..

[83] Sugiyama T., Kobayashi M., Kawamura H., Li Q., Puro D.G. (2004). Enhancement of P2X(7)-induced pore formation and apoptosis: An early effect of diabetes on the retinal microvasculature. Investig. Ophthalmol. Vis. Sci..

[84] Sugiyama T., Lee S.Y., Horie T., Oku H., Takai S., Tanioka H., Kuriki Y., Kojima S., Ikeda T. (2013). P2X(7) receptor activation may be involved in neuronal loss in the retinal ganglion cell layer after acute elevation of intraocular pressure in rats. Mol. Vis..

[85] Xia J., Lim J.C., Lu W., Beckel J.M., Macarak E.J., Laties A.M., Mitchell C.H. (2012). Neurons respond directly to mechanical deformation with pannexin-mediated ATP release and autostimulation of P2X7 receptors. J. Physiol..

[86] Niyadurupola N., Sidaway P., Ma N., Rhodes J.D., Broadway D.C., Sanderson J. (2013). P2X7 receptor activation mediates retinal ganglion cell death in a human retina model of ischemic neurodegeneration. Investig. Ophthalmol. Vis. Sci..

[87] Perez de Lara M.J., Guzman-Aranguez A., de la Villa P., Diaz-Hernandez J.I., Miras-Portugal M.T., Pintor J. (2015). Increased levels of extracellular ATP in glaucomatous retinas: Possible role of the vesicular nucleotide transporter during the development of the pathology. Mol. Vis..

[88] Stuart J.C., Linn J.G. (1985). Dilute sodium hyaluronate (Healon) in the treatment of ocular surface disorders. Ann. Ophthalmol..

[89] Johnson M.E., Murphy P.J., Boulton M. (2006). Effectiveness of sodium hyaluronate eyedrops in the treatment of dry eye. Graefes Arch. Clin. Exp. Ophthalmol..

[90] Hamano T., Horimoto K., Lee M., Komemushi S. (1996). Sodium hyaluronate eyedrops enhance tear film stability. Jpn. J. Ophthalmol..

[91] Prabhasawat P., Tesavibul N., Kasetsuwan N. (2007). Performance profile of sodium hyaluronate in patients with lipid tear deficiency: Randomised, double-blind, controlled, exploratory study. Br. J. Ophthalmol..

[92] Pauloin T., Dutot M., Warnet J.M., Rat P. (2008). In vitro modulation of preservative toxicity: High molecular weight hyaluronan decreases apoptosis and oxidative stress induced by benzalkonium chloride. Eur. J. Pharm. Sci..

[93] Rat P., Dutot M., Watzinger M., Baudouin C., Warnet J.M. (2006). Cytoprotective Effects of Four Different Hyaluronic Acids: Role of Molecular Weight. Investig. Ophthalmol. Vis. Sci..

[94] Jiang L.H. (2009). Inhibition of P2X(7) receptors by divalent cations: Old action and new insight. Eur. Biophys. J..

[95] Rassendren F., Buell G.N., Virginio C., Collo G., North R.A., Surprenant A. (1997). The permeabilizing ATP receptor, P2X7. Cloning and expression of a human cDNA. J. Biol. Chem..

[96] Steinberg T.H., Newman A.S., Swanson J.A., Silverstein S.C. (1987). ATP4-permeabilizes the plasma membrane of mouse macrophages to fluorescent dyes. J. Biol. Chem..

[97] Michel A.D., Chessell I.P., Humphrey P.P. (1999). Ionic effects on human recombinant P2X7 receptor function. Naunyn Schmiedebergs Arch. Pharmacol..

[98] Virginio C., Church D., North R.A., Surprenant A. (1997). Effects of divalent cations, protons and calmidazolium at the rat P2X7 receptor. Neuropharmacology.

[99] Falsini B., Piccardi M., Minnella A., Savastano C., Capoluongo E., Fadda A., Balestrazzi E., Maccarone R., Bisti S. (2010). Influence of saffron supplementation on retinal flicker sensitivity in early age-related macular degeneration. Investig. Ophthalmol. Vis. Sci..

[100] Piccardi M., Marangoni D., Minnella A.M., Savastano M.C., Valentini P., Ambrosio L., Capoluongo E., Maccarone R., Bisti S., Falsini B. (2012). A longitudinal follow-up study of saffron supplementation in early age-related macular degeneration: Sustained benefits to central retinal function. Evid. Based Complement. Alternat. Med..

[101] Corso L., Cavallero A., Baroni D., Garbati P., Prestipino G., Bisti S., Nobile M., Picco C. (2016). Saffron reduces ATP-induced retinal cytotoxicity by targeting P2X7 receptors. Purinergic Signal..

[102] Chew E.Y., Clemons T., SanGiovanni J.P., Danis R., Domalpally A., McBee W., Sperduto R., Ferris F.L. (2012). The Age-Related Eye Disease Study 2 (AREDS2): Study design and baseline characteristics (AREDS2 report number 1). Ophthalmology.

[103] Chew E.Y., Clemons T.E., SanGiovanni J.P., Danis R., Ferris F.L., Elman M., Antoszyk A., Ruby A., Orth D., Bressler S. (2013). Lutein + zeaxanthin and omega-3 fatty acids for age-related macular degeneration: The Age-Related Eye Disease Study 2 (AREDS2) randomized clinical trial. JAMA.

[104] SanGiovanni J.P., Agron E., Clemons T.E., Chew E.Y. (2009). Omega-3 long-chain polyunsaturated fatty acid intake inversely associated with 12-year progression to advanced age-related macular degeneration. Arch. Ophthalmol..

[105] Seddon J.M., George S., Rosner B. (2006). Cigarette smoking, fish consumption, omega-3 fatty acid intake, and associations with age-related macular degeneration: The US Twin Study of Age-Related Macular Degeneration. Arch. Ophthalmol..

[106] Hu S.J., Calippe B., Lavalette S., Roubeix C., Montassar F., Housset M., Levy O., Delarasse C., Paques M., Sahel J.A. (2015). Upregulation of P2RX7 in Cx3cr1-Deficient Mononuclear Phagocytes Leads to Increased Interleukin-1beta Secretion and Photoreceptor Neurodegeneration. J. Neurosci..

[107] Notomi S., Hisatomi T., Murakami Y., Terasaki H., Sonoda S., Asato R., Takeda A., Ikeda Y., Enaida H., Sakamoto T. (2013). Dynamic increase in extracellular ATP accelerates photoreceptor cell apoptosis via ligation of P2RX7 in subretinal hemorrhage. PLoS ONE.

[108] Notomi S., Hisatomi T., Kanemaru T., Takeda A., Ikeda Y., Enaida H., Kroemer G., Ishibashi T. (2011). Critical involvement of extracellular ATP acting on P2RX7 purinergic receptors in photoreceptor cell death. Am. J. Pathol..

[109] Guzman-Aranguez A., Perez de Lara M.J., Pintor J. (2017). Hyperosmotic stress induces ATP release and changes in P2X7 receptor levels in human corneal and conjunctival epithelial cells. Purinergic Signal..

[110] Brandle U., Kohler K., Wheeler-Schilling T.H. (1998). Expression of the P2X7-receptor subunit in neurons of the rat retina. Brain Res. Mol. Brain Res..

[111] Wheeler-Schilling T.H., Marquordt K., Kohler K., Guenther E., Jabs R. (2001). Identification of purinergic receptors in retinal ganglion cells. Brain Res. Mol. Brain Res..

[112] Yang D., Elner S.G., Clark A.J., Hughes B.A., Petty H.R., Elner V.M. (2011). Activation of P2X receptors induces apoptosis in human retinal pigment epithelium. Investig. Ophthalmol. Vis. Sci..

[113] Guha S., Baltazar G.C., Coffey E.E., Tu L.A., Lim J.C., Beckel J.M., Patel S., Eysteinsson T., Lu W., O’Brien-Jenkins A. (2013). Lysosomal alkalinization, lipid oxidation, and reduced phagosome clearance triggered by activation of the P2X7 receptor. FASEB J..

[114] Puthussery T., Fletcher E.L. (2004). Synaptic localization of P2X7 receptors in the rat retina. J. Comp. Neurol..

[115] Li A., Leung C.T., Peterson-Yantorno K., Mitchell C.H., Civan M.M. (2010). Pathways for ATP release by bovine ciliary epithelial cells, the initial step in purinergic regulation of aqueous humor inflow. Am. J. Physiol. Cell Physiol..

